# Supplementary material for: Promoter G-quadruplexes and transcription factors cooperate to shape the cell type-specific transcriptome
Source: Nat Commun. 2021 Jun 23;12:3885. doi: 10.1038/s41467-021-24198-2 (PMC8222265; doi:10.1038/s41467-021-24198-2)
Supplement: Supplementary file 1 — Supplementary Information [file 41467_2021_24198_MOESM1_ESM.pdf]

Supporting Information

Promoter G-quadruplexes and transcription factors cooperate to shape the cell type-specific transcriptome

Sara Lago[1*], Matteo Nadai[1], Filippo M. Cernilogar[2], Maryam Kazerani[2], Helena Domíniguez Moreno[2], Gunnar Schotta[2*] and Sara N. Richter[1*]

[1]Department of Molecular Medicine, University of Padua, via A. Gabelli 63, 35121 Padua, Italy.
[2] Division of Molecular Biology, Biomedical Center, Faculty of Medicine, LMU Munich, Germany.
*Corresponding authors: sara.richter@unipd.it, gunnar.schotta@med.uni-muenchen.de, sara.lago@unitn.it.

Supplementary Table 1. Comparison of pG4s found by Quadparser and G4Hunter

TOOL	G4nr	+ Strand	- Strand	Mean length (bp)
Quadparser 0-7	7414	3597	3817	24
Quadparser 0-12	10053	4981	5072	36
G4Hunter	9700	4658	5042	21

Absolute number, strand localization and mean length of the pG4s found in the BG4 immunoprecipitated peaks according to the prediction tools Quadparser (loop length 0-7 and 0-12) and G4Hunter (window size 15, score threshold 1.25).

Supplementary Table 2. List of primers and oligonucleotides used in qPCR after BG4-ChIP to test for G4 enrichment in the immunoprecipitated samples.

**Primer**	**Sequence 5’-3’**
GAPDH	FW: GCTACTAGCGGTTTTACGGGCG – RV: TGCGGCTGACTGTCGAACAGG
EIF4A1	FW: CCGGAGCGACTAGGAACTAAC – RV: GCCTTTCTTACCGGGAATCCT
MDM2	FW: GGATTTCGGACGGCTCTCG - RV: CGTTCACACTAGTGACCCGA
CDK4	FW: CCACCCTCACCATGTGACC - RV: CTTACACTCTTCGCCCTCCTC
TMCC2	FW: CCAGACACTTTGGGTGACCT – RV: AACACCTGCTCTGCCAACTT
MAP3K13	FW: GACATAGGAACGGGCAAAGA – RV: CCCATGCTGTATGTGGTCTG
ESR1	FW: GAAACAGCCCCAAATCTCAA – RV: TTGTAGCCAGCAAGCAAATG
LRRN4	FW: GAGGCTGGGATCTCAGTGTTCGG – RV: TACTCTCTGAACCAAGGGGCACT
**Oligonucleotide**	**Sequence 5’-3’**
c-myc	TGGGGAGGGTGGGGAGGGTGGGGAAGG
bcl-2	AGGGGCGGGCGCGGGAGGAAGGGGGCGGGAGCGGGGCTG
c-kit	AGGGAGGGCGCTGGGAGGAGG
scrambled	GGATGTGAGTGTGAGTGTGAGG

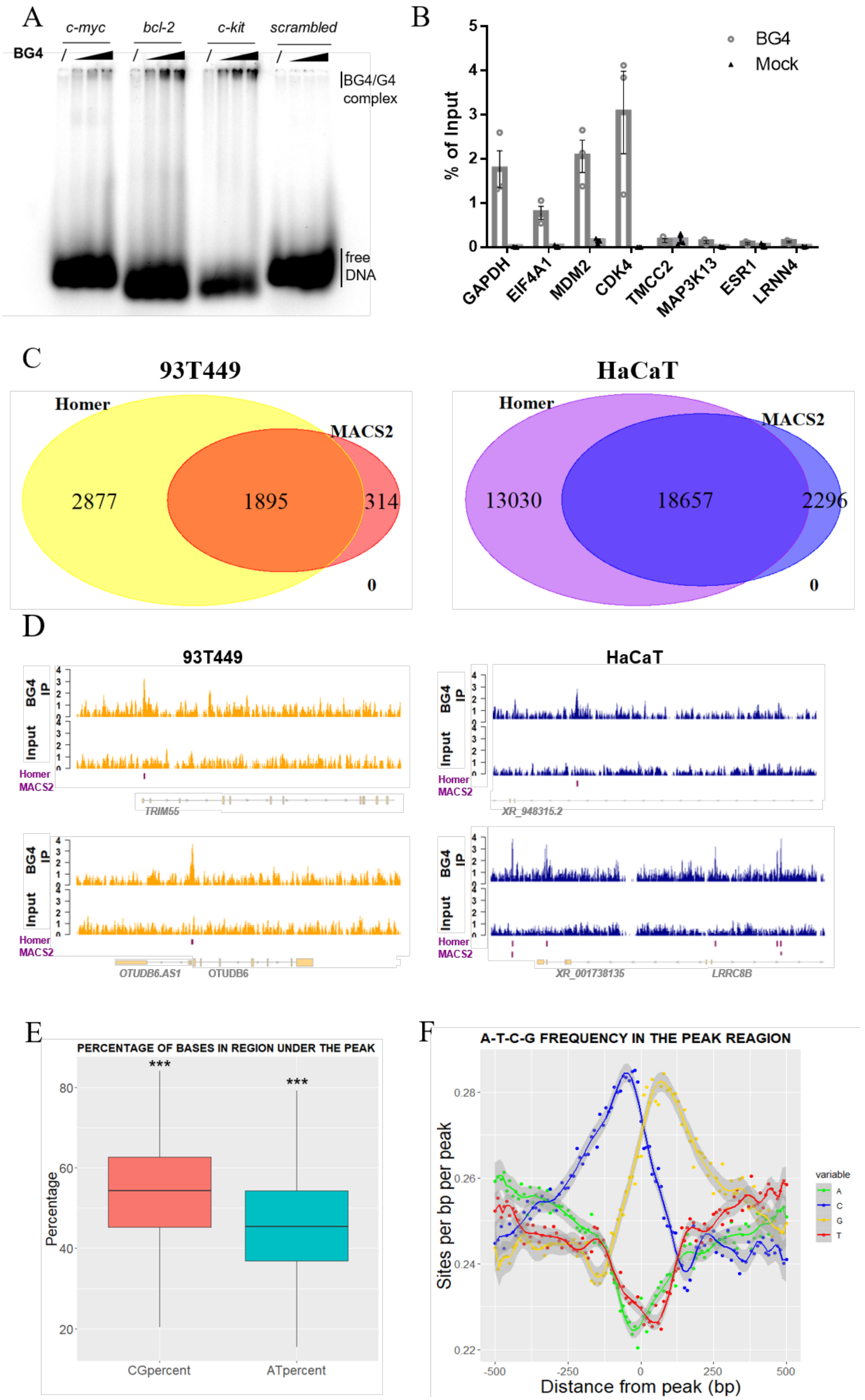

Supplementary Figure 1. ***Control parameters in the BG4 ChIP-seq analysis.*** **A)** Native EMSA experiment used to validate BG4 purified antibody capacity of binding G4 structures in a specific way. BG4 binding was measured at different antibody amounts (0 µg, 1.5 µg, 3.5 µg, 5.0

µg) in the presence of the G4 folded oligonucleotides *c-myc*, *bcl-2* and *c-kit*, and a non G4 G-rich sequence (*scrambled*) used as negative control. **B)** Control of G4 enrichment in immunoprecipitated chromatin. ChIP-qPCR on G4-positive (*GAPDH*, *EIF4A*, *MDM2*, *CDK4*) and G4-negative (*TMCC2*, *MAP3K13*, *ESR1*, *LRRN4*) control regions. Reliable G4 enrichment was observed in *GAPDH*, *EIF4A1*, *MDM2* and *CDK4* gene promoters, where G4s have been previously detected[4]. These were chosen as *GAPDH* and *EIF4A* preserve a conserved expression along different cell lines[6,7], while *MDM2* and *CDK4* are highly expressed relevant oncogenes in WDLPS carcinogenesis[8,9]. In contrast, negligible G4 signal was obtained in *TMCC2*, *MAP3K13*, *ESR1* and *LRRN4* gene promoters, the sequences of which do not contain any G4s. Grey bars represent the BG4 immunoprecipitated regions, while black bars correspond to the sample immunoprecipitated in the absence of BG4 antibody. Bars correspond to the mean fold enrichment over Input, reported as percentage of Input. Value corresponding to three individual biological replicates are reported, together with S.E.M. **C and D)** Comparison of G4-peaks calling by mean of Homer and MACS2 tools, where **C)** Venn diagram showing peaks intersection of peaks called by mean of Homer and MACS2 for 93T449 (left) and HaCaT cell lines (right). Peaks intersection was calculated by mean of ChIPpeakAnno package of R [1,2] . **D)** Genomic view of G4-ChIP-seq peaks that were identified by mean of Homer software but not by mean of MACS2. Two example regions are shon for 93T449 (orange tracks – left) and HaCaT cell lines (blue tracks – right). BG4 IP and Input tracks are shown for each region. The purples annotations on the bottom of each graph span the region of peaks as identified by mean of the two tools Homer and MACS2, as indicated on the left side. **E)** Percentage of CG and AT bases in BG4 immunoprecipitated peaks. Asterisks represent the significance level calculated from a two-sided T-test (C.I. 95%) comparing the CG and AT content in BG4 ChIP peaks with respect to the genomic abundance of the corresponding bases (i.e. an average of 41% for CG and 59% for AT) [3]. *** corresponds to p-value < 0.001. **F)** A, T, C, G base frequency in BG4 immunoprecipitated peaks reported as function of the distance from the peak centre.

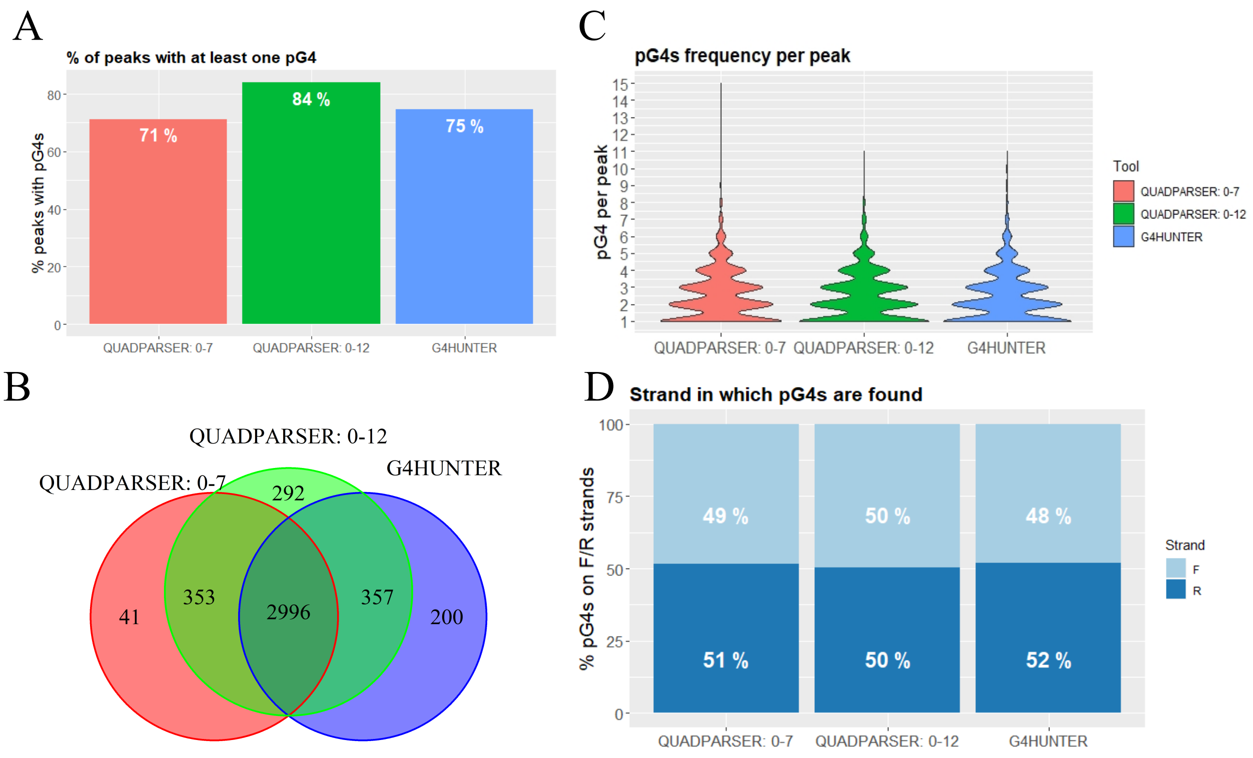

Supplementary Figure 2. Computational analysis of putative G4s (pG4s) in BG4 ChIP-seq peaks. A) Percentage of peaks containing at least one pG4 according to Quadparser and G4Hunter computational prediction tools that identify canonical G4s based on a regular-expression-matching algorithm and canonical/non-canonical G4s based on a sliding window algorithm, respectively. Quadparser prediction was performed with two different settings: G4 loop-length in the range 0-7 and in the range 0-12. For G4Hunter prediction, the window size was set to 20 and the score threshold at 1.25. **B)** Venn diagram comparing the peaks in which at least one pG4 was found by Quadparser (loops length 0-7 or 0-12) and G4Hunter (window 20 and score threshold 1.25) **C)** Frequency plot of the amount of pG4s found in each ChIP-seq peak by Quadparser (loops length 0-7 or 0-12) and G4Hunter (window 20 and score threshold 1.25). **D)** Percentage of pG4s found on the forward and reverse strand according to Quadparser (loop length 0-7 or 0-12) and G4Hunter (window size 20, score threshold 1.25).

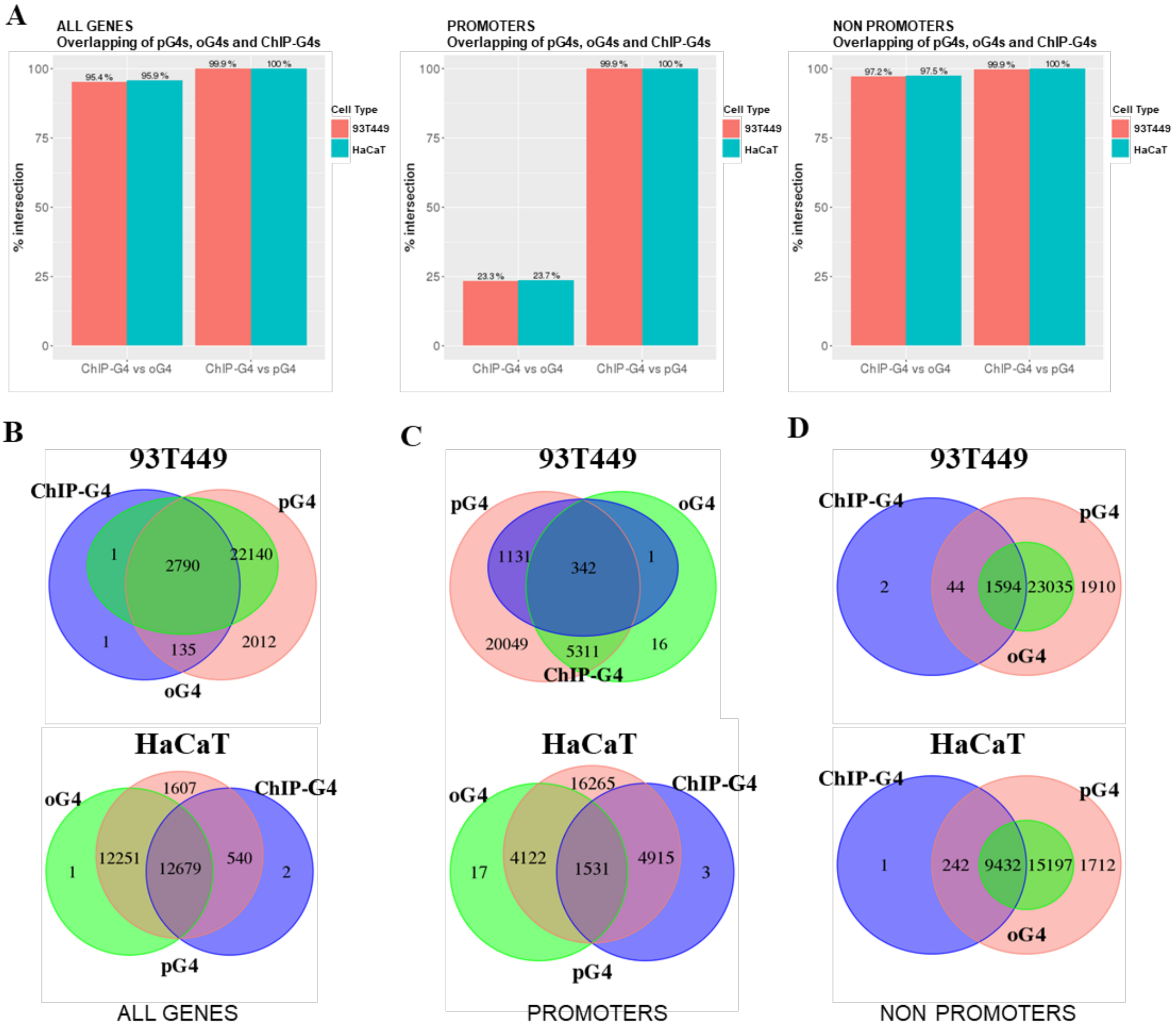

Supplementary Figure 3. ***Overlapping of pG4s, oG4s and BG4 ChIP-G4s*.** **A)** Percentage of regions in which BG4 ChIP-G4s overlap with pG4s as calculated by Quadparser, considering G-tracts of 2 or more Gs and loops of 0-12 nts, or with oG4s determined by G4-seq method. The calculation was performed both for 93T449 and HaCaT cells. From left to right, we considered all G4-containing gene, genes with G4s in their promoter, genes with G4s elsewhere than the promoter. **B)** Venn diagram showing overlapping of pG4s, oG4s and ChIP-G4s in all G4 containing genes for 93T449 (upper panel) and HaCaT (lower panel). **C)** Venn diagram showing overlapping of pG4s, oG4s and ChIP-G4s in G4 containing genes at their promoter for 93T449 (upper panel) and HaCaT (lower panel). **D)** Venn diagram showing overlapping of pG4s, oG4s and ChIP-G4s in G4 containing genes outside the promoter for 93T449 (upper panel) and HaCaT (lower panel).

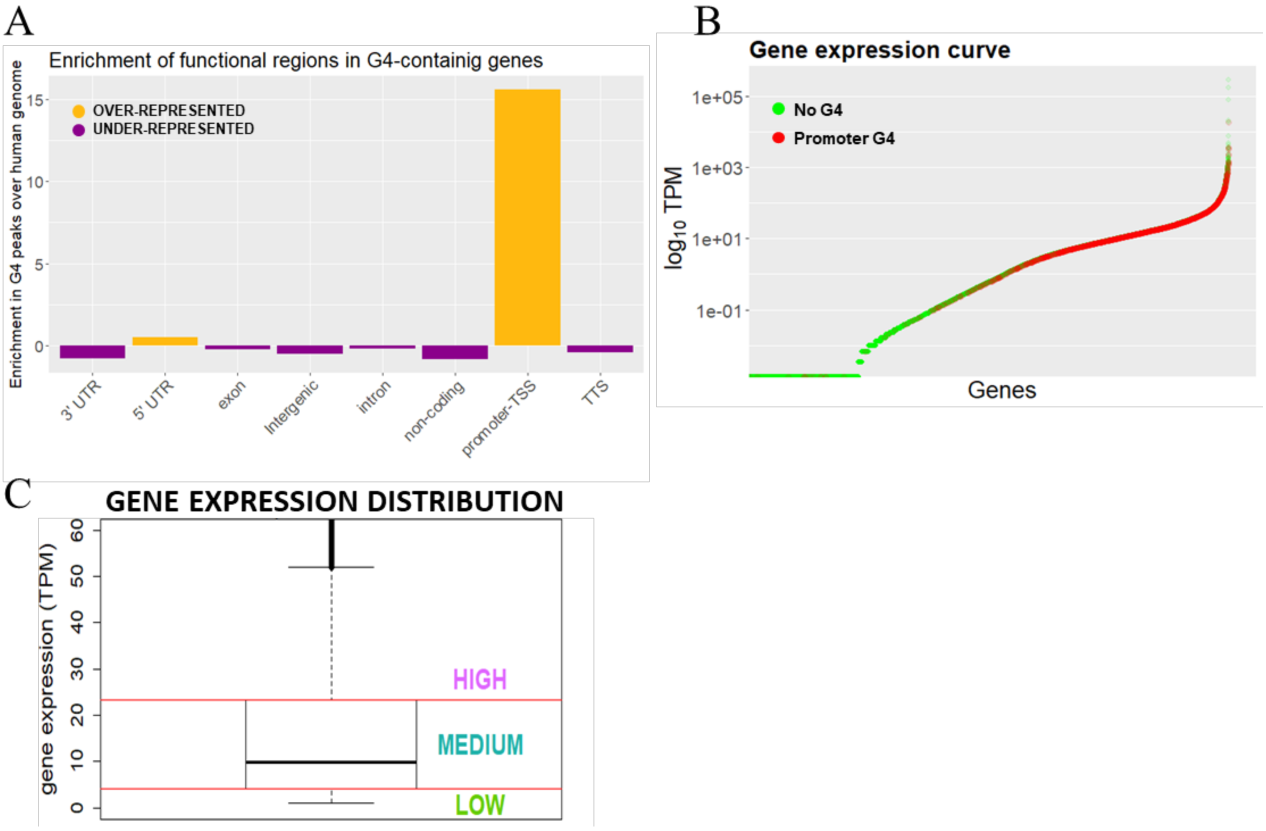

Supplementary Figure 4. ***G4 peaks distribution in the genome.*** **A)** Enrichment of functional regions in G4-containing peaks. Orange bars correspond to positively enriched regions, purple bars to under-represented regions. The relative enrichment was calculated as (functional region frequency in G4-ChIP peaks - functional region frequency in whole genome)/ functional region frequency in whole genome. Enrichment of 0 means that the frequency of the functional region in the human genome and in ChIP-peaks is the same, which is equivalent to a random representation. Negative enrichment corresponds to under-representation, while positive enrichment to over-representation. **B)** A curve representing gene expression of all 93T449 genes is represented, where green dots are genes for which no promoter G4 was found and red dots are genes with G4 at their promoter. Gene expression level is reported in TPM and log10 scale on the y axis. **C)** Gene expression distribution of all the expressed genes (at least one transcript per gene) in 93T449 cells according to the RNA-seq data. Three expression categories were defined based on the expression distribution quartiles: the first quartile corresponded to low expression, the central two quartiles corresponded to medium expression and the upper quartile corresponded to high expression.

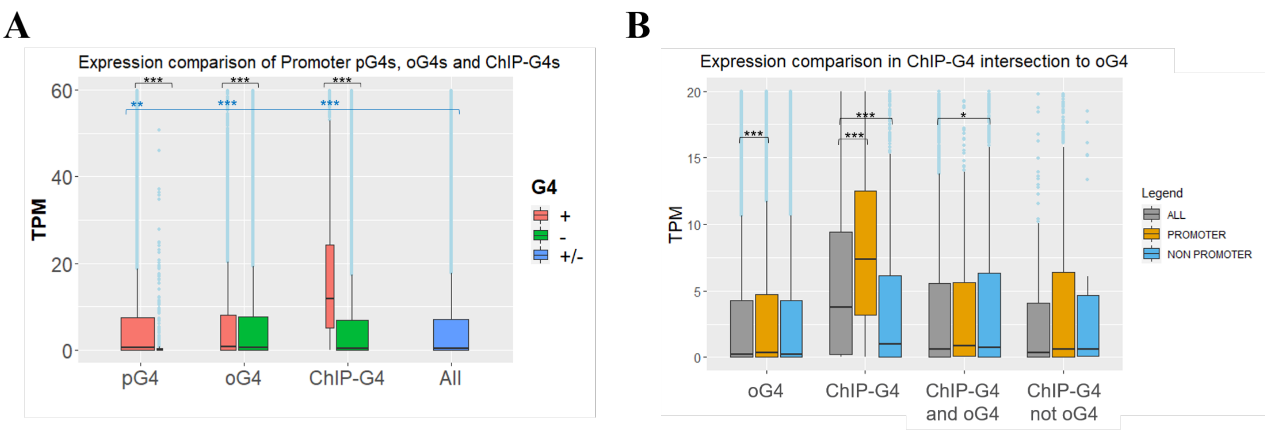

Supplementary Figure 5. ***Expression comparison of genes with pG4s, oG4s and ChIP-G4s in 93T449 cells.*** **A)** Expression distribution of genes with (+) or without (-) pG4s, oG4s and ChIP-G4s compared to all genes (+/-). Both expressed and non-expressed (< 1 transcript per gene) were considered. Box widths are proportional to the numerosity of each category. Asterisks indicate significance level, calculated according to two-sided T-test (C.I. 95%), where *** = p-value < 0.001 and ** = p-value < 0.01. Black asterisks indicate comparisons between elements within groups (i.e. genes with + and without – G4s), while blue asterisk indicate comparisons of G4 positive (+) genes of each group with respect to *All* genes category. **B)** Expression distribution of genes with oG4s, ChIP-G4s their intersection (ChIP-G4 and oG4) and genes residing outside of the intersection (ChIP-G4 not oG4s). Genes in each category were further subdivided according to the position of the detected G4 (promoter, non promoter) or considered independently of the G4 position. Asterisks indicate significance level, calculated according to two-sided T-test (C.I. 95%), where *** = p-value < 0.001 and * = p-value < 0.05.

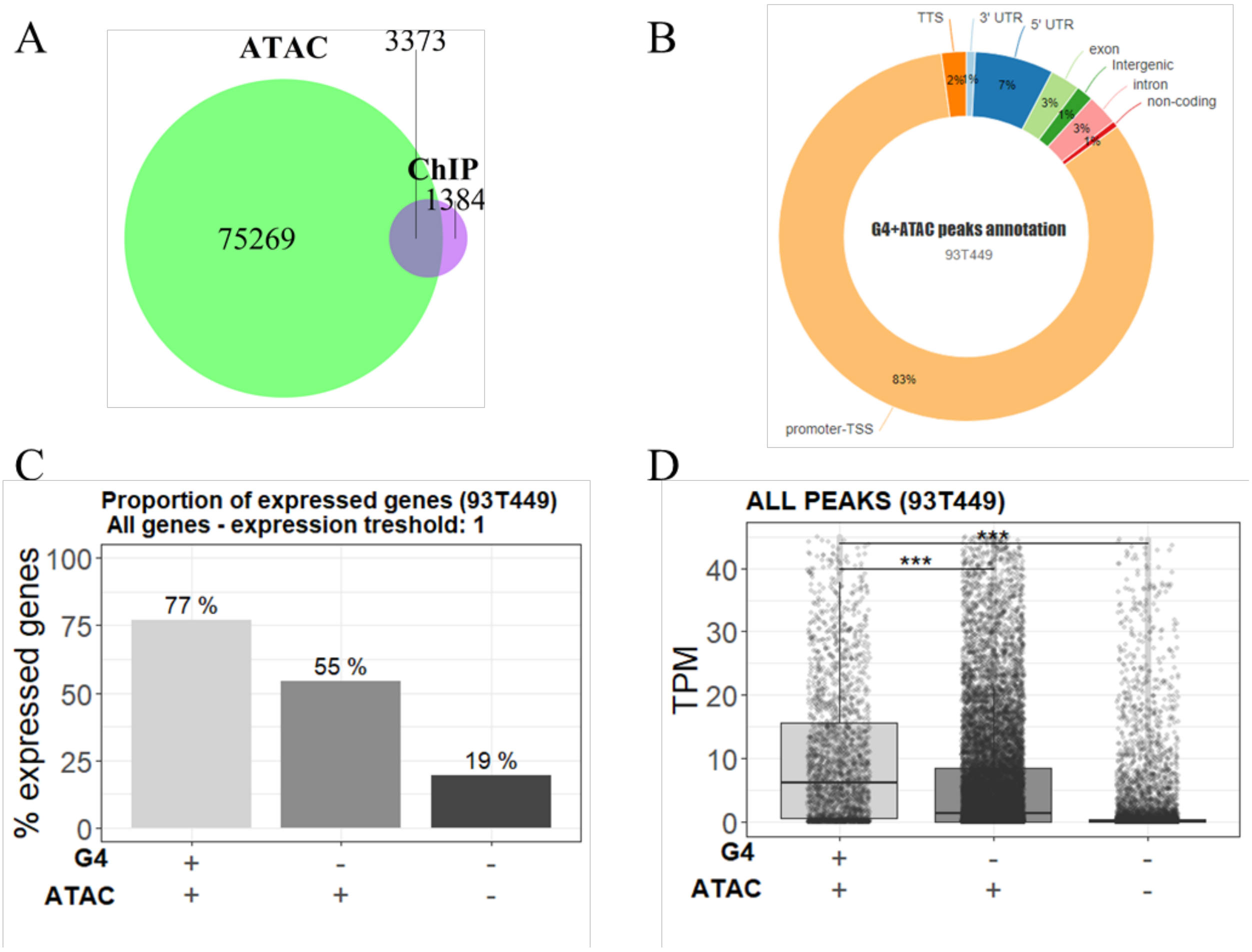

Supplementary Figure 6. ***Integration of G4 ChIP-seq, ATAC-seq and RNA-seq data.*** **A)** Venn diagram displaying the intersection between peak regions corresponding to immunoprecipitated G4s (violet) and open chromatin regions (light blue) mapped by ATAC-seq. **B)** Percentage distribution of G4 peaks that overlap with ATAC-seq peaks in functional genomic regions according to HOMER gene annotation. Percentages are normalized over the genomic abundance of each functional region. **C)** Percentage proportion of expressed genes grouped according to the presence of G4s and open chromatin signal in any functional region of the gene. Expression threshold was set to one transcript per gene. **D)** Expression distribution of all genes grouped according to the presence of G4s and open chromatin signal in any position. Expression threshold was set to 1 transcript per gene. T-test was applied to measure statistical significance (*** = pval 0.001).

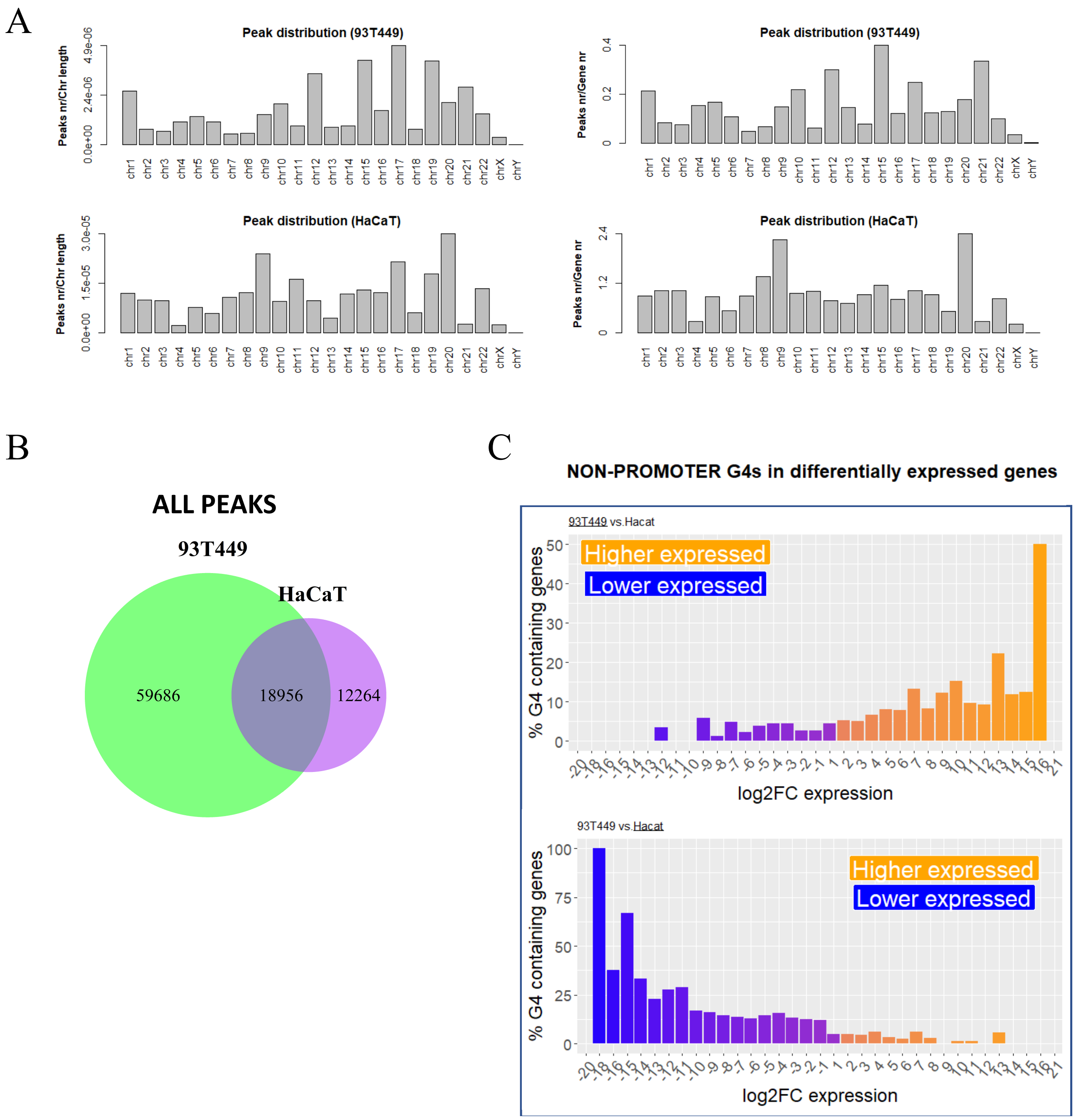

Supplementary Figure 7. Comparison of genome-wide G4 distribution, open chromatin regions and gene expression between 93T449 and HaCaT cells. A) Distribution of G4 peaks along chromosomes, normalized either by the chromosome length (left panels) and the number of genes encoded in each chromosome (right panels). **B)** Venn diagram showing the intersection of ATAC-seq peaks between 93T449 and HaCaT. **C)** Percentage of genes containing at least one G4 only outside of their promoter in 93T449 (upper panel) or HaCaT (lower panel) cells, in function of their differential expression in the two cell lines.

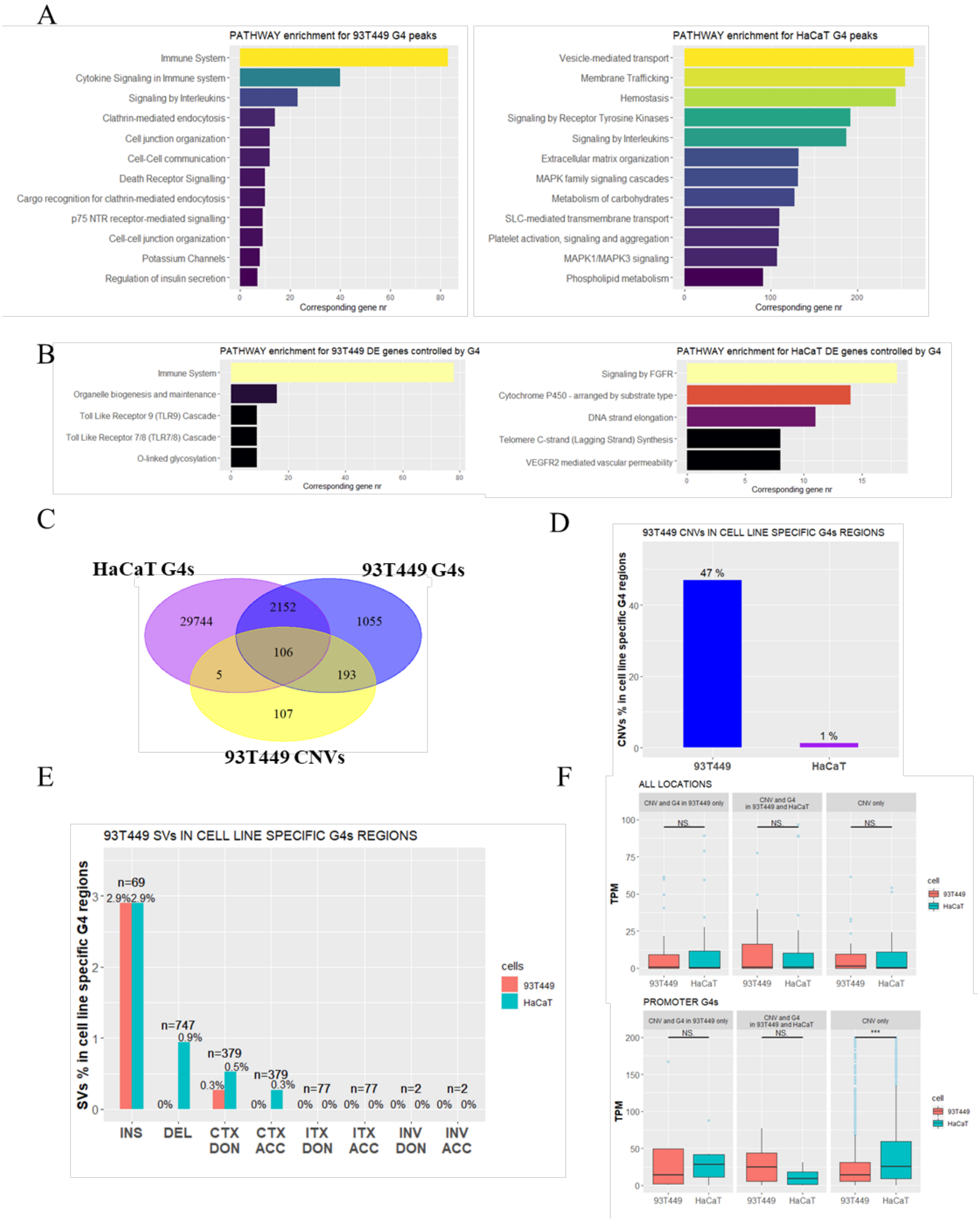

Supplementary Figure 8. Pathway enrichment, CNVs and SVs analysis on G4 containing genes for 93T449 and HaCaT cells. A) Pathway enrichment analysis for genes harboring G4s specifically in 93T449 (left) or HaCaT cells (right) independently of the G4 position in the gene and the corresponding gene expression level. P-value ≤ 0.01. **B)** Pathway enrichment analysis for differentially expressed genes harboring G4s. Genes with significantly higher expression in 93T449 (left) or HaCaT cells (right) and harboring a G4 were considered in the calculation. P-value ≤ 0.01. **C)** Venn diagram showing the intersection of BG4 G4 ChIP-seq peaks for 93T449

(blue) and HaCaT (purple) cell lines with 93T449 CNVs detected by WGS (yellow). **D)** Bar plot displaying the percentage of 93T449 CNVs overlapping with regions of cell line specific G4s (i.e., G4s that were detected by BG4 ChIP-seq only in one of the two cell lines). **E)** Bar plot showing the percentage of 93T449 SVs overlapping with regions of cell line-specific G4s (i.e., G4s that were detected by BG4 ChIP-seq only in one of the two cell lines). INS=insertion, DEL=deletion, CTX DON= inter chromosomal translocation donor region, CTX ACC= inter chromosomal translocation acceptor region, ITX DON= intra chromosomal translocation donor region, ITX ACC=intra chromosomal translocation acceptor region, INV DON= inversion donor region, ITX ACC=inversion acceptor region. The group total numerosity is reported above each bar group. **F)** Box plot showing the gene expression distribution (TPM) of genes which have 93T449-specific CNVs and: G4s in 93T449 only, G4s both in 93T449 and HaCaT, or CNVs and no G4 in 93T449. For each group the expression of the same genes in 93T449 and HacaT cells in shown. The upper graph considered genes independently from the G4 location, while the lower graph considered only genes with promoter G4s i 93T449. Statistical significance of the comparisons was calculated by two-sided T-test (C.I. 95%), asterisks corresponding to significance p-value are reported on the graph: *** p-value < 0.001, NS indicates that the statistical difference is not significant.

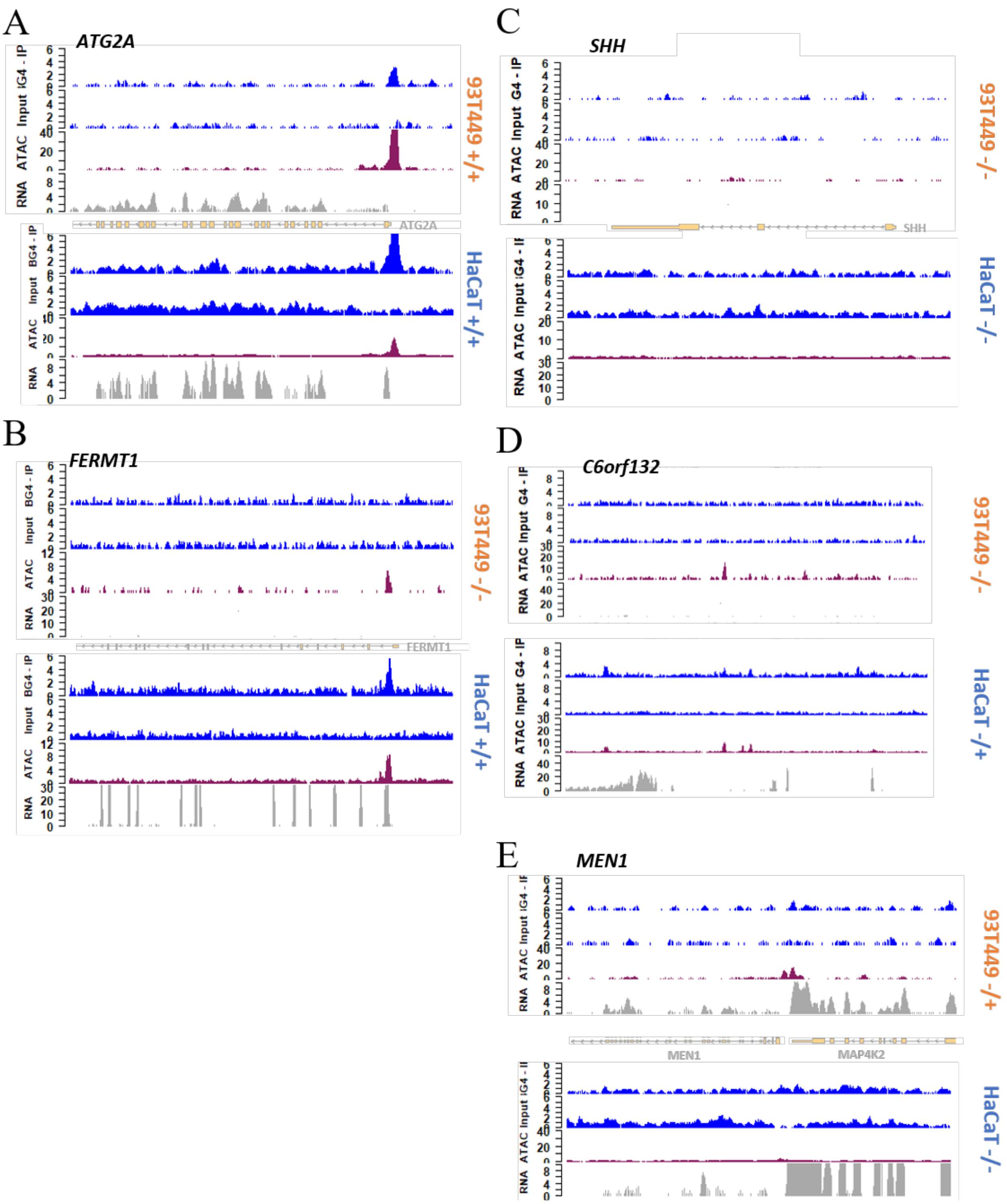

Supplementary Figure 9. Genomic view of example regions showing how different combinations of open chromatin (ATAC) and G4 (BG4-IP) result in different transcriptional outputs (RNA) in 93T449 and HaCaT cells. A) *ATGA2* gene, with open chromatin and G4 at its promoter in both 93T449 and HaCaT cells; **B)** *FERMT1* gene, with open chromatin and G4 at its promoter only in HaCaT cells; **C)** *SHH* gene, showing no open chromatin nor G4 in both 93T449 and HaCaT cells; **D)** *C6orf132* gene, showing open chromatin but not G4 only in HaCaT cells and **E)** *MEN1* gene, showing open chromatin but not G4 only in 93T449 cells.

A

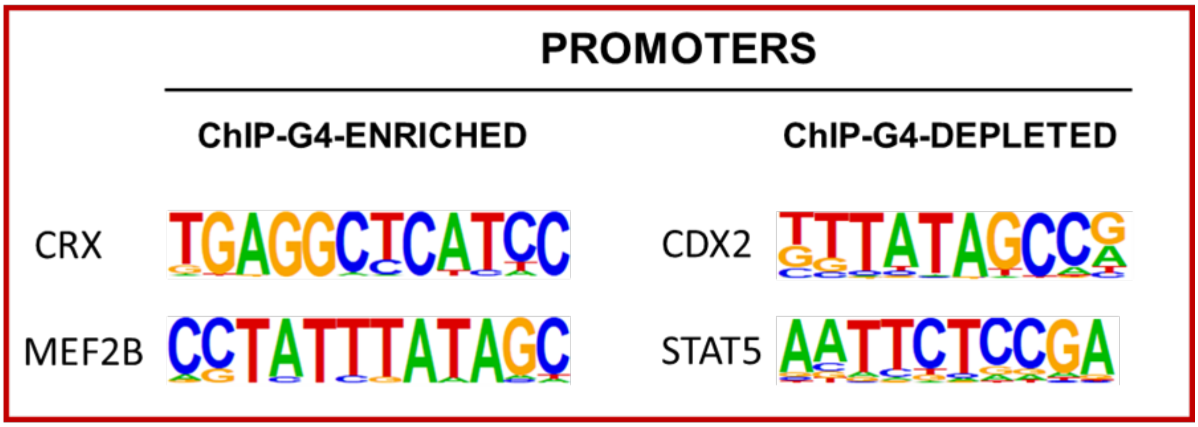

B

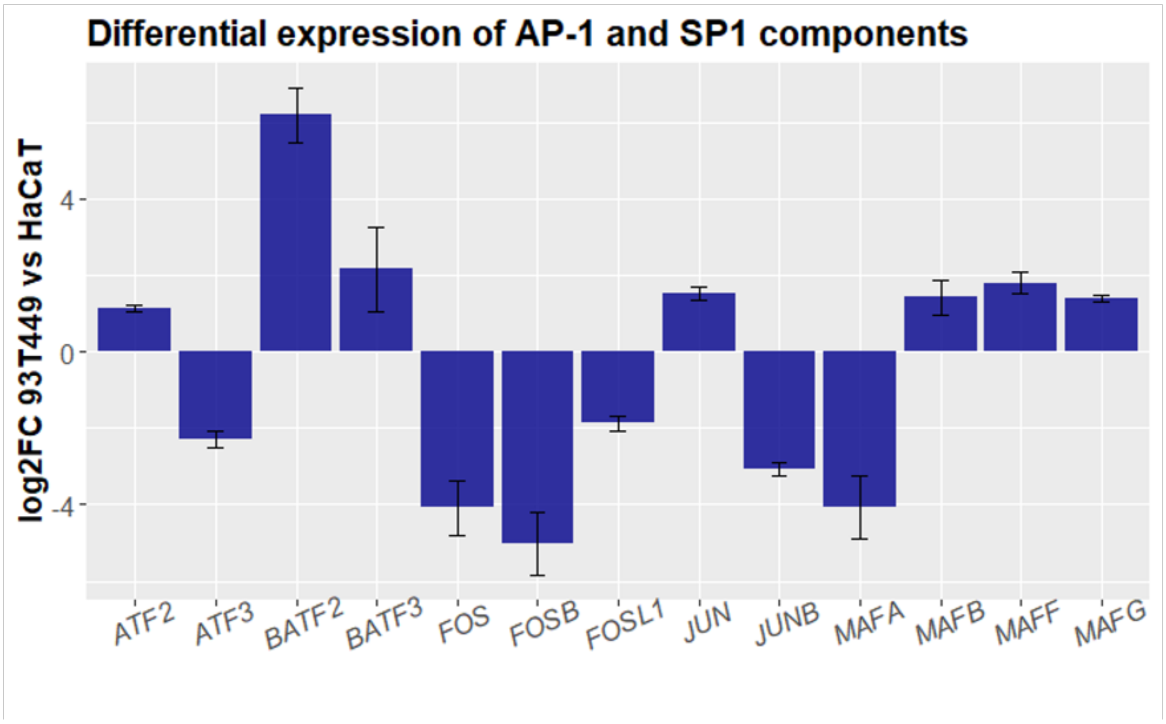

Supplementary Figure 10. Analysis of AP-1 and Sp1 component expression and TFBS enrichment in ChIP-G4-enriched and depleted promoters. A) Significantly represented motifs for TFs were calculated by HOMER software in the extended promoter region (-1000 to + 750 from TSS) of the ChIP-G4s enriched (left) or depleted (right) genes. CRX (percentage of target genes 2 %, percentage of background 0.18 %) and MEF2B (percentage of target genes 1.57 %, percentage of background 0.11 %) were identified in ChIP-G4s enriched promoters; while CDX2 (percentage of target genes 6.43 %, percentage of background 4.22 %) and STAT5 (percentage of target genes 12.80 %, percentage of background 9.77 %) were identified in promoters of ChIP-G4s depleted genes. **B)** Differential expression of AP-1 and SP1 protein components in 93T449 and HaCaT cells. Error bars are log2FC standard errors among replicates. Positive values represent genes with higher expression in 93T449, while negative values are genes with higher expression in HaCaT.

Supplementary References

1. Zhu, L. J. *et al.* ChIPpeakAnno: a Bioconductor package to annotate ChIP-seq and ChIP-chip data. *BMC Bioinformatics* **11**, 237 (2010).
2. Zhu, L. J. Integrative analysis of ChIP-chip and ChIP-seq dataset. *Methods Mol Biol* **1067**, 105–124 (2013).
3. Motulsky, A. G. History of Human Genetics*. in *Vogel and Motulsky's Human Genetics* (eds. Speicher, M. R., Motulsky, A. G. & Antonarakis, S. E.) 13–29 (Springer, 2010). doi:10.1007/978-3-540-37654-5_2.
