# Supplementary material for: Promoter G-quadruplexes and transcription factors cooperate to shape the cell type-specific transcriptome
Source: Nat Commun. 2021 Jun 23;12:3885. doi: 10.1038/s41467-021-24198-2 (PMC8222265; doi:10.1038/s41467-021-24198-2)
Supplement: Supplementary file 3 — Description of Additional Supplementary Files [file 41467_2021_24198_MOESM3_ESM.pdf]

Description of Additional Supplementary Files

File Name: Supplementary Data 1
Description: Table of annotated peaks for G4 ChIP-seq in 93T449 cells, merge of 2 replicates.

File Name: Supplementary Data 2
Description: Table with TPM gene expression from 3 biological replicates of RNA-seq in 93T449 cells.

File Name: Supplementary Data 3
Description: Table of annotated Omni-ATAC-seq peaks for 93T449 cells.

File Name: Supplementary Data 4
Description: Table with TPM gene expression from 4 biological replicates of RNA-seq in HaCaT cells.

File Name: Supplementary Data 5
Description: Table of annotated ATAC-seq peaks for HaCaT cells, merge of 2 replicates.

File Name: Supplementary Data 6
Description: Table of annotated peaks for G4 ChIP-seq in HaCaT cells, merge of 2 replicates.

File Name: Supplementary Data 7
Description: Table of significant differentially expressed genes between 93T449 and HaCaT cell lines, considering 3 and 4 biological replicates respectively.

File Name: Supplementary Data 8
Description: list and logos of de novo and known transcription factor binding motifs in G4 peaks for 93T449 cells.
