# Supplementary material for: Promoter G-quadruplexes and transcription factors cooperate to shape the cell type-specific transcriptome
Source: Nat Commun. 2021 Jun 23;12:3885. doi: 10.1038/s41467-021-24198-2 (PMC8222265; doi:10.1038/s41467-021-24198-2)
Supplement: Supplementary file 12 — Reporting Summary [file 41467_2021_24198_MOESM12_ESM.pdf]

nature research

Corresponding author(s): Sara N. Richter, Gunnar Schotta, Sara Lago

Last updated by author(s): Jun 3, 2021

# Reporting Summary

Nature Research wishes to improve the reproducibility of the work that we publish. This form provides structure for consistency and transparency in reporting. For further information on Nature Research policies, see our Editorial Policies and the Editorial Policy Checklist.

## Statistics

For all statistical analyses, confirm that the following items are present in the figure legend, table legend, main text, or Methods section.

| n/a | Confirmed | |
|---|---|---|
| ☐ | ☒ | The exact sample size (*n*) for each experimental group/condition, given as a discrete number and unit of measurement |
| ☐ | ☒ | A statement on whether measurements were taken from distinct samples or whether the same sample was measured repeatedly |
| ☐ | ☒ | The statistical test(s) used AND whether they are one- or two-sided<br>*Only common tests should be described solely by name; describe more complex techniques in the Methods section.* |
| ☒ | ☐ | A description of all covariates tested |
| ☒ | ☐ | A description of any assumptions or corrections, such as tests of normality and adjustment for multiple comparisons |
| ☐ | ☒ | A full description of the statistical parameters including central tendency (e.g. means) or other basic estimates (e.g. regression coefficient) AND variation (e.g. standard deviation) or associated estimates of uncertainty (e.g. confidence intervals) |
| ☐ | ☒ | For null hypothesis testing, the test statistic (e.g. $F$, $t$, $r$) with confidence intervals, effect sizes, degrees of freedom and $P$ value noted<br>*Give P values as exact values whenever suitable.* |
| ☒ | ☐ | For Bayesian analysis, information on the choice of priors and Markov chain Monte Carlo settings |
| ☒ | ☐ | For hierarchical and complex designs, identification of the appropriate level for tests and full reporting of outcomes |
| ☒ | ☐ | Estimates of effect sizes (e.g. Cohen's *d*, Pearson's *r*), indicating how they were calculated |

*Our web collection on statistics for biologists contains articles on many of the points above.*

## Software and code

Policy information about availability of computer code

| | |
|---|---|
| Data collection | Illumina HiSeq 1500 platform was employed for sequencing data collection.<br>Typhoon FLA 9000 software was used for EMSA data acquisition.<br>Uvitec Software was used for Western Blot data acquisition.<br>Nikon NIS Element software v4.0 was used for Immunofluorescence image acquisition.<br>ImageJ v1.53c was used for immunofluorescence data quantification.<br>GraphPad Prism v6 was used for plotting data. |
| Data analysis | Raw FASTQ reads were trimmed to remove adaptor contamination and aligned to the human reference genome version hg38 using Bowtie1 (http://bowtie-bio.sourceforge.net/index.shtml). Reads with more than 2 mismatches and multimapped reads were excluded from further analysis. G4–ChIP peaks were mapped using HOMER software v4.10 http://homer.ucsd.edu/homer/index.html) considering only peaks with at least 2 fold more normalized tags count in the target experiment with respect to the input (used as control), disabled local tag count and poisson p-value threshold of 0.0001. Homer software v4.10 was also used for peak annotation and transcription factor binding sites identification within G4 ChIP-seq peaks. ATAC-seq peaks were also called by mean of Homer software with default parameter.<br>RNA–seq reads were aligned to the human reference genome (hg30 - GRCh38) with TopHat v2.1.1 and filtered by using samtools v1.6 to remove alignments with quality lower than 20, not primary alignments and PCR duplicates. Differential gene expression was performed using the Bioconductor package DESeq2 v1.20.0 and 'apeglm' for LFC shrinkage (s-value < 0.1; fold change > 1.0).<br>All further analysis were performed by mean of R v3.6.2 and associated packages, the main of which are cited in the methods section of the manuscript. |

For manuscripts utilizing custom algorithms or software that are central to the research but not yet described in published literature, software must be made available to editors and reviewers. We strongly encourage code deposition in a community repository (e.g. GitHub). See the Nature Research guidelines for submitting code & software for further information.

## Data

Policy information about availability of data

All manuscripts must include a data availability statement. This statement should provide the following information, where applicable:

- Accession codes, unique identifiers, or web links for publicly available datasets
- A list of figures that have associated raw data
- A description of any restrictions on data availability

All genomic data produced in the present project (93T449 G4-ChIP-seq, ATAC-seq and RNA-seq) have been deposited in the NCBI GEO database under accession number GSE145543 (reviewer token: wzwvciymxvqrlal). HaCaT cells datasets for G4-ChIP-seq, ATAC-seq and RNA-seq were instead downloaded from GEO at the following accession number GSE76688. The oG4s dataset produced in the presence of K+ was retrieved from GEO at the following accession number GSE63874. The list of CNVs and SVs for 93T449 cells obtained by BIC-seq and WGS respectively are available as online supplementary material to the paper ref doi:10.1534/genetics.117.300552.
The validated target genes of AP-1 and SP1 were retrieved from ENCODE database (https://www.encodeproject.org/), accessed through Harmonizome92 (https://maayanlab.cloud/Harmonizome/). Reactome database (https://reactome.org/) was employed to calculate significant pathway enrichment. The complete list of human genes annotated on the GRCh38-hg38 reference genome was retrieved from BioMart Ensembl database ( HYPERLINK "http://www.ensembl.org/biomart/martview/365b1f5efbf012a182846ce458b6537a" http://www.ensembl.org/biomart/martview).

# Field-specific reporting

Please select the one below that is the best fit for your research. If you are not sure, read the appropriate sections before making your selection.

☒ Life sciences ☐ Behavioural & social sciences ☐ Ecological, evolutionary & environmental sciences

For a reference copy of the document with all sections, see nature.com/documents/nr-reporting-summary-flat.pdf

# Life sciences study design

All studies must disclose on these points even when the disclosure is negative.

| | |
|---|---|
| Sample size | No statistical calculation of sample number was performed. The number of replicates for sequencing experiments was based on the numbers used in previous publications of papers in the same field (G-quadruplex sequencing; ChIP-seq, ATAC-seq, RNA-seq) and published in high impact factor journals following ENCODE guidelines: Nat Genet 48, 1267–1272 (2016) https://doi.org/10.1038/ng.3662; Nat Struct Mol Biol 25, 951–957 (2018). https://doi.org/10.1038/s41594-018-0131-8.<br><br>For IF experiments 74-150 cells were measured, to account for a statistically relevant population (Nat Struct Mol Biol 27, 424–437 (2020). https://doi.org/10.1038/s41594-020-0408-6). |
| Data exclusions | No data were excluded form the analysis. |
| Replication | Reproducibility of experimental data was assessed by comparison of the concordance among technical and biological repilcates at different stages during sample preparation. Reproduciblity and robustness of data analysis was assessed by comparing results obtained by mean of different tools, when possible. ChIP-qPCR was performed in two independent biological replicates, each account of 2-3 technical replicates, all successful. RNA-seq was performed in 3 independent biological replicates, each successful. ATAC-seq was performed once. IF experiments were perfomed in two independent biological replicates, 5-9 fields of the same replicate were measured separately to amek the statistics. Each replicate was successful and gave consistent results. Co-IP was performed at least 3 times, all successful. |
| Randomization | Randomization was not necessary since each condition was treated independently and no sample/cell/component was excluded from the analysis, implying that no random choice was done. |
| Blinding | Blinding was not relevant in this study setting as there is no possible influence of the investigator on groups allocation. In fact, all experiments and data analysis were performed in the same experimental conditions. |

# Reporting for specific materials, systems and methods

We require information from authors about some types of materials, experimental systems and methods used in many studies. Here, indicate whether each material, system or method listed is relevant to your study. If you are not sure if a list item applies to your research, read the appropriate section before selecting a response.

## Materials & experimental systems

| n/a | Involved in the study | |
|---|---|---|
| ☐ | ☒ | Antibodies |
| ☐ | ☒ | Eukaryotic cell lines |
| ☒ | ☐ | Palaeontology and archaeology |
| ☒ | ☐ | Animals and other organisms |
| ☒ | ☐ | Human research participants |
| ☒ | ☐ | Clinical data |
| ☒ | ☐ | Dual use research of concern |

## Methods

| n/a | Involved in the study | |
|---|---|---|
| ☐ | ☒ | ChIP-seq |
| ☒ | ☐ | Flow cytometry |
| ☒ | ☐ | MRI-based neuroimaging |

# Antibodies

| | |
|---|---|
| Antibodies used | BG4 antibody used for G4-ChIP-seq was expressed and purified in BL21(DE3) competent cells from the BG4-encoding plasmid (kindly provided by Professor Shankar Balasubramanian, University of Cambridge, UK), detailed procedure is described in the manuscript methods section.<br>Commercial BG4 used for Immunofluorescence (IF) was purchased from Sigma-Aldrich (#MABE917).<br>Secondary antibody used for IF is anti-mouse Alexa 488 (A21204, ThermoFisher).<br>M2 anti-FLAG (F3165, Sigma Aldrich) was used for ChIP-seq, Westrn Blot and IF.<br>For co-IP followed by Western Blot were used anti-AP-1 Thermo Scientific™ #MA5-15172 (clone C.238.2), anti-Sp1 ChIPAb+™ Merck #17-601, anti-FLAG Sigma Aldrich #F3165 Sigma-Aldrich. Secondary antibodies used in co-IP Western Blots were secondary goat anti-rabbit (Merck-Millipore #12-34) and goat anti-mouse (Merck-Millipore #12-349) HRP antibodies.<br>The used antibody dilutions are reported in the methods section of the manuscript. |
| Validation | In house produced BG4 antibody was validated in vitro by mean of Western Blot with anti-FLAG epitope tag antibody (F3165, Sigma Aldrich) and Electrophoretic Mobility Shift Assay (EMSA) to test its activity and specificity towards the binding to G4 folded oligonucleotides and absence of binding to non-G4 DNA sequences, as similarly reported in previous publications (https://doi.org/10.1038/nchembio.2228; DOI: 10.1371/journal.pone.0158794).<br>The commercial Anti-DNA-G-quadruplex structures, clone BG4 antibody (Cat. No. MABE917) was resolved by SDS-PAGE, transferred to PVDF membrane and probed with Anti-FLAG epitope tag antibody (MAB3118)(https://www.merckmillipore.com/IT/it/product/Anti-DNA-G-quadruplex-structures-Antibody-clone-BG4,MM_NF-MABE917; https://www.ncbi.nlm.nih.gov/pmc/articles/PMC6391399/).<br>Anti-mouse Alexa 488 (A21204, ThermoFisher) quantum yield determined relative to fluorescein at pH 8.0. Activity verified by cell staining performed using a primary antibody specific for cell type and isotype, and this product (https://www.thermofisher.com/antibody/product/Rabbit-anti-Mouse-IgG-H-L-Cross-Adsorbed-Secondary-Antibody-Polyclonal/A-21204; doi: 10.1083/jcb.201911129).<br>anti-Sp1 ChIPAb+™ Merck #17-601 was produced under quality system ISO 9001 (https://www.merckmillipore.com/IT/it/product/ChIPAb+-Sp1-ChIP-Validated-Antibody-and-Primer-Set,MM_NF-17-601; doi: 10.1016/j.jcmgh.2019.10.005), verified by mean of ChIP-qPCR and Western Blot.<br>Quality assurance for goat anti-rabbit (Merck-Millipore #12-348; https://www.merckmillipore.com/IT/it/product/Goat-Anti-Rabbit-IgG-Antibody-HRP-conjugate,MM_NF-12-348; doi: 10.1038/s41467-018-06091-7) and goat anti-mouse (Merck-Millipore #12-349 (https://www.merckmillipore.com/IT/it/product/Goat-Anti-Mouse-IgG-Antibody-HRP-conjugate,MM_NF-12-349; doi: 10.1093/hmg/ddz095) HRP antibodies was<br>evaluated by Western Blot. |

# Eukaryotic cell lines

Policy information about cell lines

| | |
|---|---|
| Cell line source(s) | 93T449 (ATCC® CRL-3043™) |
| Authentication | ATCC comprehensively perform authentication and quality-control tests on all distribution lots of cell lines. |
| Mycoplasma contamination | All the used cell lines tested negative for mycoplasma contamination. |
| Commonly misidentified lines<br>(See ICLAC register) | None of the used cell lines is in the Register of Misidentified cell lines |

# ChIP-seq

## Data deposition

☒ Confirm that both raw and final processed data have been deposited in a public database such as GEO.

☒ Confirm that you have deposited or provided access to graph files (e.g. BED files) for the called peaks.

| | |
|---|---|
| Data access links<br>*May remain private before publication.* | All genomic data produced in the present project (93T449 G4-ChIP-seq, ATAC-seq and RNA-seq) have been deposited in the NCBI GEO database under accession number GSE145543 (reviewer token: wzwvciymxvqrlal) |

Files in database submission

GSM4320546 IP-93T449-BG4_rep1
GSM4320547 INPUT-93T449-BG4_rep1
GSM4320548 IP-93T449-BG4_rep2
GSM4320549 INPUT-93T449-BG4_rep2

Genome browser session
(e.g. UCSC)

https://www.genome.ucsc.edu/cgi-bin/hgTracks?db=hg38&lastVirtModeType=default&lastVirtModeExtraState=&virtModeType=default&virtMode=0&nonVirtPosition=&position=chr12%3A57744843%2D57755156&hgsid=1050198207_p1TdPlZ3GkGa1OKaAR2cqdOWaeIn

## Methodology

Replicates

Two independent biologiacal replicates were performed for ChIP-seq data comprising of IP and Input samples. Both replicates account for 2 to 4 technical replicates. Goodness of replicates was assessed by mean of qPCR for estimating enrichment of positive and negative control targets, both before and after library preparation. Concordancy of the sequenced replicates was assessed by PCA (Principal Component Analysis) clustering and comparison of identified peaks.

Sequencing depth

All ChIP-seq experiments were sequenced using 30 Million reads depth with single-end 50 bp chemistry.

Antibodies

BG4 antibody used for G4-ChIP-seq was expressed and purified in BL21(DE3) competent cells from the BG4-encoding plasmid (kindly provided by Professor Shankar Balasubramanian, University of Cambridge, UK), detailed procedure is described in the manuscript methods section. BG4 antibody was validated in vitro by mean of Western Blot with anti-FLAG epitope tag antibody (F3165, Sigma Aldrich) and Electrophoretic Mobility Shift Assay (EMSA) to test its activity and specificity towards the binding to G4 folded oligonucleotides and absence of binding to non-G4 DNA sequences.
M2 anti-FLAG (F3165, Sigma Aldrich) was used to capture chromatin-bound BG4 on magnetic beads for ChIP procedure.

Peak calling parameters

Reads mapping was performed by mean of bowtie tool excluding multimapped reads and using human genome hg38 (GRCh38) indices with the following command: bowtie -q -n 2 --best --chunkmbs 2000 -p 32 -m 1 -S  $indices $fastqfile.
The output s.sam file was used to generate a tag directory to allow Homer to perform peak calling.
Peak calling was performed by mean of HOMER software with the following command and parameters: findPeaks <IP_file> -o <output_file> -style factor -i <input_file> -F 2 -L 0

Data quality

Quality of sequencing data was evaluated by mean of FastQC tool, considering the percentage of duplicates, percentage of GC content, Mean Quality Scores, Adapter content and overrepresented sequences. Visual evaluation of peaks and comparison with previously published datasets GSE76688. Different peak calling parameters were tested to ensure the better condition for reliable peaks identification: G4–ChIP peaks  were mapped using HOMER http://homer.ucsd.edu/homer/index.html) considering only peaks with at least 2 fold more normalized tags count in the target experiment with respect to the input (used as control), disabled local tag count and poisson p-value threshold of 0.0001.
A summary of peak calling parameter for both G4 ChIP-seq replicates is reported below:
HOMER Peaks
# Peak finding parameters:
# tag directory = IP_BG4_93T_m1
#
# total peaks = 3867
# peak size = 175
# peaks found using tags on both strands
# minimum distance between peaks = 350
# fragment length = 174
# genome size = 2000000000
# Total tags = 32924725.0
# Total tags in peaks = 161065.0
# Approximate IP efficiency = 0.49%
# tags per bp = 0.015759
# expected tags per peak = 2.758
# maximum tags considered per bp = 1.0
# effective number of tags used for normalization = 10000000.0
# Peaks have been centered at maximum tag pile-up
# FDR rate threshold = 0.001000000
# FDR effective poisson threshold = 2.374038e-07
# FDR tag threshold = 15.0
# number of putative peaks = 28142
#
# input tag directory = Input_BG4_93T_m1
# Fold over input required = 2.00
# Poisson p-value over input required = 1.00e-04
# Putative peaks filtered by input = 24273
#
# size of region used for local filtering = 10000
# Poisson p-value over local region required = 1.00e-04
# Putative peaks filtered by local signal = 0
#
# Maximum fold under expected unique positions for tags = 2.00
# Putative peaks filtered for being too clonal = 2
#

# cmd = findPeaks IP_BG4_93T_m1 -o GS404_IP_BG4_93T_m1_F2L0_peaks.txt -style factor -i Input_BG4_93T_m1 -F 2 -L 0

HOMER Peaks
# Peak finding parameters:
# tag directory = IP_BG4_93T_rep2_m1
#
# total peaks = 1335
# peak size = 164
# peaks found using tags on both strands
# minimum distance between peaks = 328
# fragment length = 164
# genome size = 2000000000
# Total tags = 30942187.0
# Total tags in peaks = 83683.0
# Approximate IP efficiency = 0.27%
# tags per bp = 0.014512
# expected tags per peak = 2.380
# maximum tags considered per bp = 1.0
# effective number of tags used for normalization = 10000000.0
# Peaks have been centered at maximum tag pile-up
# FDR rate threshold = 0.001000000
# FDR effective poisson threshold = 2.356008e-07
# FDR tag threshold = 14.0
# number of putative peaks = 27436
#
# input tag directory = Input_BG4_93T_rep2_m1
# Fold over input required = 2.00
# Poisson p-value over input required = 1.00e-04
# Putative peaks filtered by input = 26100
#
# size of region used for local filtering = 10000
# Poisson p-value over local region required = 1.00e-04
# Putative peaks filtered by local signal = 0
#
# Maximum fold under expected unique positions for tags = 2.00
# Putative peaks filtered for being too clonal = 1
#
# cmd = findPeaks IP_BG4_93T_rep2_m1 -o GS430_IP_BG4_93T_rep2_m1_F2L0_peaks.txt -style factor -i Input_BG4_93T_rep2_m1 -F 2 -L 0

Software

HOMER software was employed for ChIP-seq peak calling and annotation and identification of enriched transcriptiona factors binding sites within called peaks. MACS2 was also used for peak calling for comparison and quality evaluation. All further analysis on the identified peaks were conducted by mean of custom R code or using public R packages. All the scripts are available upon request.
